# Spatial Analysis of the Home Addresses of Hospital Patients with Hepatitis B Infection or Hepatoma in Shenzhen, China from 2010 to 2012

**DOI:** 10.3390/ijerph110303143

**Published:** 2014-03-14

**Authors:** Tao Hu, Qingyun Du, Fu Ren, Shi Liang, Denan Lin, Jiajia Li, Yan Chen

**Affiliations:** 1 School of Resources and Environmental Science, Wuhan University, Wuhan 430079, China; E-Mails: hutao.cumt@163.com (T.H.); qydu@whu.edu.cn (Q.D.); 2 Key Laboratory of Geographic Information Systems, Ministry of Education, Wuhan University, Luoyu Road 129, Wuhan 430079, China; 3 Shenzhen Center for Health Information, Renmin Road North 2210, Luohu District, Shenzhen 518001, China; E-Mails: ldn308@163.com (D.L.), jiajiali831@gmail.com (J.L.), chy@newhealth.com.cn (Y.C.)

**Keywords:** geographic information system, spatial analysis, hepatitis, hepatoma

## Abstract

*Background*: Hepatoma associated with hepatitis B infection is a major public health problem in Shenzhen (China) and worldwide. China has the largest number of people infected with the hepatitis B virus (HBV), and many studies have demonstrated that HBV infection is associated with hepatoma development. Shenzhen officials have been attempting to monitor and control these diseases for many years. The methodology and the results of this study may be useful in developing a system to monitor, prevent and control these diseases. *Methods*: The aim of the study was to analyze HBV infection and hepatoma distribution characteristics and patterns in Shenzhen by combining geographic information system (GIS) technology and spatial analysis. The study used data from patients at the district level from the 2010–2012 population censuses. *Results*: Only one-third of the patients were female, and 70.7% of all cases were 20–50 years of age. There was no global spatial correlation of the distribution of hepatitis B infections and hepatomas; however, there was a local spatial correlation, and certain sub-districts of the Nanshan district had significant agglomeration effects. Based on incidence density and rate maps, we can conclude that the Shenzhen special zone had a higher incidence density and rate of hepatitis B infections and hepatomas compared with the area outside of the Shenzhen special zone during 2010–2012. *Conclusions*: This study demonstrated substantial geographic variation in the incidence of hepatitis B infection and hepatoma in Shenzhen. The prediction and control of hepatitis B infections and hepatoma development and interventions for these diseases should focus on disadvantaged areas to reduce disparities. GIS and spatial analysis play an important role in public health risk-reduction programs and may become integral components in the epidemiologic description, analysis and risk assessment of hepatitis B and hepatoma.

## 1. Introduction

China has the largest number of people infected with hepatitis B virus (HBV) worldwide and more than one-third of the World’s 350 to 400 million chronic HBV carriers have lived in China [1]. Hepatitis B remains a major public health and social problem, although the prevalence has been decreasing. The positivity rate for hepatitis B virus surface antigen (HBsAg) in the general population decreased from 9.75% during 1992–1995 to 7.18% in 2006, and the percent positive for children under the age of five has decreased to below 1%, which approaches the rates in Europe and North America [2]. Many studies have demonstrated that HBV is one of the 17 important pathogens associated with hepatoma. The modes of transmission of HBV are similar to those of acquired immune deficiency syndrome (AIDS) and include sexual contact, blood transmission and maternal-neonatal transmission. While implementing strict disease control, the Chinese government has ordered that individuals and companies cannot test for HBV during recruiting, and no company can refuse to hire staff because of HBV positivity, as HBV appears to not be transmitted through general contact.

Geographic information systems (GIS) can be used to conduct spatial analyses of regional data. Because approximately 80% of epidemiological data have spatial properties, GIS and spatial analysis have become innovative tools and important components of public health and epidemiological studies [3,4,5,6,7,8]. Over the past 20 years, the rapid development of GIS has facilitated the examination of spatial patterns and processes [9]. With GIS, it is possible to link the data of interest with background variables, such as census, socio-economic and environmental data on the population, according to the map coordinates [10,11]. GIS has become a tool for health evaluation and health risk assessment, decision support and prevention planning [12]. 

Hepatoma is one of the most frequent malignant tumors in general and the most common liver malignancy because of its high incidence in many populous countries [13,14,15,16]. Significant differences in hepatoma incidence are found across nine geographic regions within the United States [17]. Recently, hepatoma has gained attention as a public health issue in China. 

Depending on the quantity and quality of the data and the methodology used in the analysis, a given map may be useful for the health sector and decision makers [10]. The health sector can use spatial analysis to take steps to prevent and control the spread of disease [18]. This study used GIS to establish a geo-database of Shenzhen hepatitis B cases and hepatoma cases in hospitals from 2010 to 2012; the spatial distribution characteristics of the patients were analyzed to provide a scientific basis for the prevention and control of the diseases.

This paper is organized as follows: Section 2 briefly introduces the study area and methods and the relevant data for Shenzhen; Section 3 describes the results for the spatial distribution characteristics and trends; and Section 4 draws conclusions and discusses potential future studies.

## 2. Methods

### 2.1. Study Area

**Figure 1:**
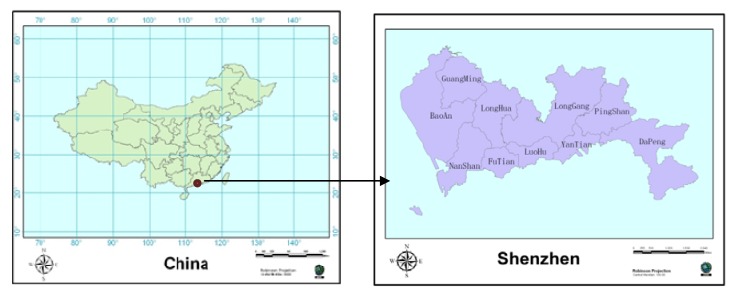
The location of Shenzhen City in China; the names of districts in Shenzhen City.

Shenzhen is located south of the Tropic of Cancer, from 113°46' to 114°37' east longitude and between 22°27′ and 22°52′ northern latitude, in the southeast coastal areas of Guangdong and northeast of the Pearl River Estuary (Figure 1). Shenzhen is 81.4 km from east to west and 10.8 km from north to south, covering a total area of 1,952 km^2^, and has a population of more than ten million individuals. Shenzhen is connected with Hong Kong’s New Territories south of the Shenzhen River and Shenzhen Bay, with Dongguan to the north and with Huizhou to the east. One of the earliest special economic zones in China was in Shenzhen (including the four districts of Luohu, Futian, Nanshan and Yantian). At present, there are a total of 10 districts (Luohu, Futian, Nanshan, Yantian, Baoan, Longgang, Guangming, Pingshan, Longhua and Dapeng) in Shenzhen. Figure 1 shows the location of Shenzhen City in China and the names of the districts in Shenzhen City not shown.

### 2.2. Data

Home address data were obtained from the medical records of hospital patients with acute hepatitis B, chronic hepatitis B or hepatoma in Shenzhen during 2010 to 2012; the data were stored in the Shenzhen Center for Health Information (SCHI). The population data for different municipal administrative regions and functional areas were also obtained from the SCHI, an institute directly administered by the Health, Population and Family Planning Commission of Shenzhen Municipality.

Administrative data for the division of Shenzhen were obtained from the Urban Planning, Land and Resources Commission of the Shenzhen Municipality.

### 2.3. Methods

This study used the methods of spatial agglomeration, spatial autocorrelation analysis [19], spatial hotspots analysis, incidence density and incidence rate analysis. The hotspot analysis calculates the Getis-Ord 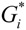 statistic [20,21] for each disease case in Shenzhen during 2010–2012 to distinguish either high or low cluster values spatially with ArcGIS 10. The Getis-Ord local statistic is given as follows:

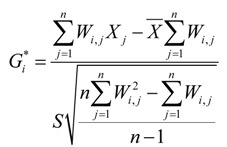
(1)
Where *x_j_* is the number of cases in region *j*, *w_i,j_* is the spatial weight between regions *i* and *j*, n is the total number of cases, *X* is the average number of cases, and *S* is the mean square error. The 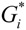 statistic is a z-score, so no further calculations are required. The hotspot map can identify significant spatial clusters of high values (hot spots) and low values (cold spots). Spatial cluster analysis tests the spatial distribution of disease patterns in a particular geographical environment, with the cluster coefficient *C = S*^2^*/X*, where *X* is the average number of cases in each area, for the corresponding variance. If *C* < 1, then the spatial distribution of the cases is uniform; if *C* = 1, then the spatial distribution is random, and if *C* > 1, then the spatial distribution of the cases represents an aggregated distribution. In local Moran’s I analysis, COType fields with significantly (*p* < 0.05) high levels of clustering are expressed as HH, and those with significantly (*p* < 0.05) low levels of clustering are expressed as LL. If the Z-score is <1.96, then there is a significant level (*p* < 0.05) of spatial outliers. The output elements for the classification field for COType indicate whether high value elements and surrounding elements are lower (HL), or whether low elements and surrounding elements are high value elements (LH).

Incidence density analysis and incidence rate analysis are the two basic and important methods of epidemiologic study. Disease mapping aims to analyze the geographical distribution of disease, searching for the high risk area of the disease on the basis of hypothesis of pathogenic factors. It is widely used in the field of public health and environmental health. The incidence rates were calculated using 2010–2012 census data and inter-census population estimates at the sub-district level. For the purpose of analysis, the study period was divided into three intervals based on trends in incidence: year 2010; year 2011; and year 2012. The study area was divided into the ten areas illustrated in Figure 1. 

## 3. Results and Discussion

**Figure 2:**
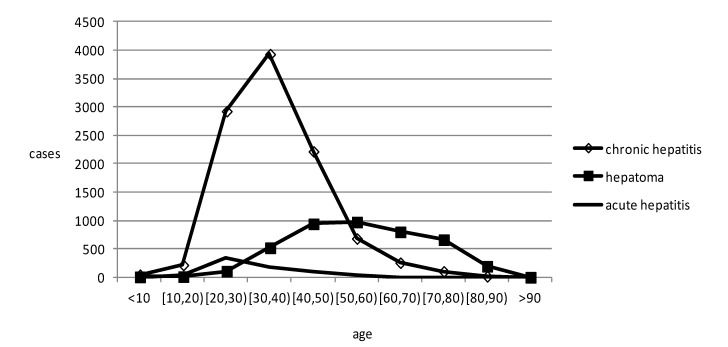
The age distribution of hepatitis B and hepatoma in Shenzhen from 2010 to 2012.

For the hepatitis B and hepatoma analysis study, there were a total of 15,352 cases from 2010 to 2012. Basic information on the cases including home address, onset, hospital treatment, age and sex were used to obtain accurate and reliable geographic epidemiology data on hepatitis B infection and hepatoma incidence. There were a total of 11,785 male patients and 3,567 female patients; therefore, the number of male patients was three times the number of female patients. Figure 2 shows the age distribution of hepatitis B and hepatoma in Shenzhen from 2010 to 2012; the average age of the patients was 41.1 years, and 70.7% were 20–50 years of age. The most common age ranges for the occurrence of chronic hepatitis B, hepatoma and acute hepatitis B were 20–50 years old, 40–60 years old and 20–30 years old, respectively. 

**Figure 3:**
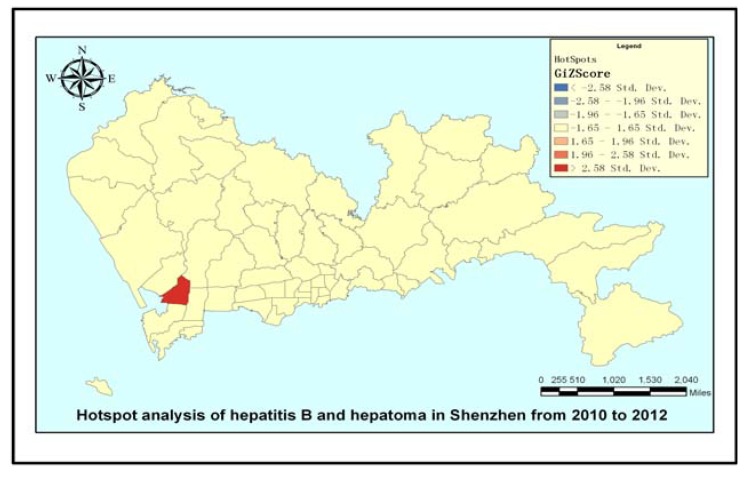
Hotspot analysis of hepatitis B and hepatoma in Shenzhen.

**Figure 4:**
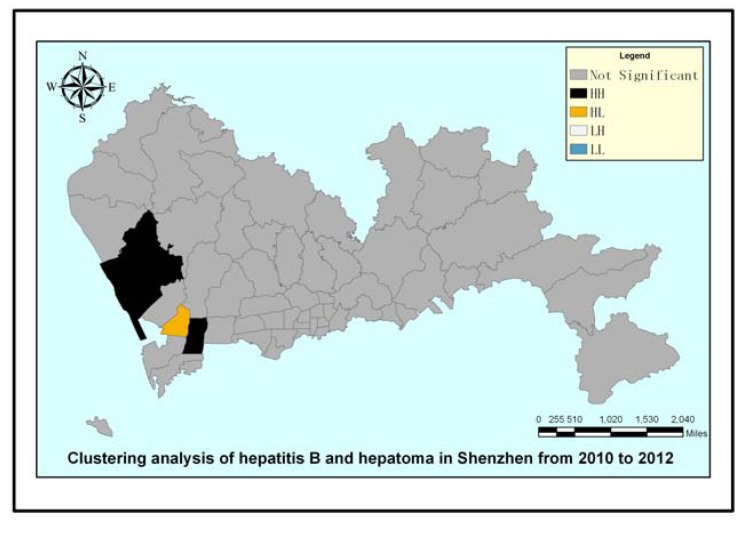
Clustering analysis of hepatitis B and hepatoma in Shenzhen.

The map in Figure 3 displays a hotspot analysis for hepatoma in Shenzhen from 2010 to 2012 at the sub-district level. We use hot spot analysis (Getis-ord 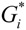) tool in ArcGIS software to draw a hot spot map. The only red region is the Nantou sub-district, which was identified as having a significantly high value. The map reflects hepatoma cases gathered in the Nantou sub-district in the geographical space scale. In the map of Figure 4, the black regions of the Xixiang sub-district and Yuehai sub-district have significant levels of high value (HH) clustering, whereas the yellow region of the Nantou sub-district is mainly composed of significantly high values of low around the outliers (HL) clustering. The other sub-districts showed no significant spatial agglomeration effect. Based on Figure 3 and Figure 4, we can conclude that the Nanshan district had a higher hepatoma incidence rate than other districts in Shenzhen during 2010–2012. Prevention and intervention measures should focus on these areas for its agglomeration; we should pay more attention to these areas in the health resource allocation. To a great extent, medical convenient depends on the spatial location and distribution of medical services.

Spatial autocorrelation analysis was used to determine that Moran’s Index = 0.016505; given the z-score of 0.99, the pattern does not appear to differ significantly from random. Spatial autocorrelation analysis shows that the distribution of hepatoma had no global spatial correlation, whereas Figure 4 shows that the Xixiang sub-district, Yuehai sub-district and Nantou sub-district in Shenzhen had a local spatial correlation through ArcGIS software. 

**Figure 5:**
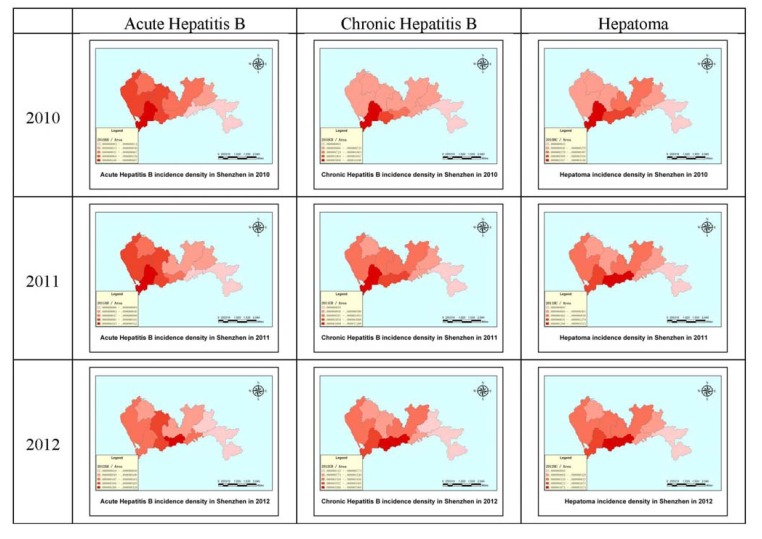
The incidence densities of acute hepatitis B, chronic hepatitis B and hepatoma in Shenzhen in 2010, 2011 and 2012.

Figure 5 demonstrates how the incidence density of acute hepatitis B, chronic hepatitis B and hepatoma at the district level in Shenzhen changed during the period 2010–2012. The depth of the color represents the density, with deeper colors indicating higher incidence densities. Overall, the incidence density is higher in the south than in the north and higher in the west than in the east. The Nanshan district had the highest incidence density of hepatitis B and hepatoma cases in 2010, whereas the Luohu and Futian districts had the highest levels in 2012. The Dapeng new district had the lowest incidence density during 2010–2012; the large area and small population of Dapeng new district may be an important reason for this result. There is a notable trend that the incidence density moved from west to east. From 2010 to 2012, the incidence density of the three diseases decreased each year.

**Figure 6:**
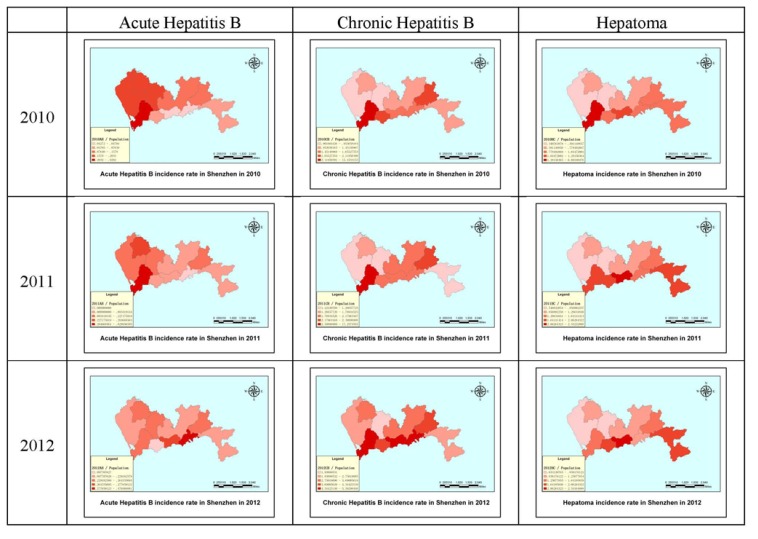
The incidence rates of hepatitis B and hepatoma in Shenzhen in 2010, 2011 and 2012.

The disease incidence rate is the number of new cases per population in a given time period. As in Figure 5, the depth of the color represents density in Figure 6, with deeper colors indicating higher incidence rates. The incidence rate in the Nanshan district was higher than the other districts for all disease types in 2010, whereas the Baoan district and the Longhua new district had the lowest incidence rates for chronic hepatitis and hepatoma. The Luohu and Yantian districts had the lowest incidence rates of acute hepatitis in 2010. In 2011, the Nanshan district still had the highest incidence rate for acute and chronic hepatitis, but the Luohu district had the highest incidence rate of hepatoma. Compared with 2010, the areas with the lowest densities of disease remained basically unchanged in 2011. The 2012 map shows that the Nanshan district had the highest incidence rate of chronic hepatitis, whereas the Yantian and Luohu districts had the highest incidence rates of the other two diseases. We can clearly see that between 2010 and 2012, the disease incidence was in transition from west to east, which can provide the scientific basis for disease prevention and intervention in Shenzhen city. The incidence rates of the three diseases decreased significantly year to year.

## 4. Conclusions

The power of GIS for spatial data management and analysis for disease prevention and for enabling the provision of technical support and allocation of health resources through geographical data collection and spatial analysis has been demonstrated [19]. This study estimated the spatial agglomeration effects of hepatitis B infection and hepatoma development. The results show that the distribution of acute hepatitis, chronic hepatitis and hepatoma had certain spatial agglomeration effects in Shenzhen during the period 2010–2012. High incidence areas are mostly middle-aged, and low incidence areas are mostly young maybe a possible reason for the local clustering. Further research is required to determine the possible causes for the agglomeration effects [22]. 

There was no global spatial autocorrelation; however, there was local spatial autocorrelation at the sub-district level for the distribution of hepatitis B infections and hepatoma cases. The statistical correlations between proximal cases of hepatitis B and hepatoma were derived and verified by simulation of the distributions of hepatitis B and hepatoma [23]. Ord and Getis [24] provide a statistical test for local spatial autocorrelation in the presence of the global autocorrelation that is characteristic of heterogeneous spatial data.

A gender difference for these diseases is quite apparent, not only in Shenzhen but also in China and worldwide. Although women in Shenzhen had an overall lower risk of hepatitis B infection and hepatoma than men, their associated relative risk was higher, and the number of other risk factors identified for women in our analysis was fewer. Other epidemiological studies of hepatitis B infection and hepatoma have often omitted women or have included them in numbers too small for separate analysis. Gender differences in hepatitis B and hepatoma mortality and risk factors are substantial and warrant further study [25]. This study found that 70.7% of the patient cases were 20–50 years of age, which may strongly relate to the high prevalence of smoking and drinking alcohol [26,27,28,29,30] in this age group. Harland and Austin [29] found evidence of a strong, significant and apparently super-multiplicative effect of heavy smoking and heavy drinking in the development of hepatoma. Chi-Ting Chiang [31] also found that elevated concentrations of heavy metals in the soil are somewhat correlated to the levels of exposure, which may promote hepatitis B and hepatoma development in local residents. The main difficulty is indeterminacy, as the relationships between individuals of different ages were not found [32].

Based on the incidence density and rate maps, we can conclude that the Shenzhen special zone had a higher incidence density and rate of hepatitis B infection and hepatoma than outside of the Shenzhen special zone from 2010 to 2012. Maps are fundamental tools for detecting abnormal spatial patterns of health outcomes and associations with environmental risk factors; therefore, the importance of spatial analysis techniques and methods will increase. Both the disease incidence density and rate had a notable trend of transfer from west to east. GIS spatial analysis technology can be used to analyze and evaluate hepatitis and hepatoma incidence, which can be used to predict local laws of distribution; however, the characteristics of the population rather than the geographical environment itself determines the aggregation of epidemics and outbreaks. To further improve the accuracy of analysis and forecasting, research on the factors that may influence disease should increase, and comprehensive consideration should be given to the social population, social environment, season, weather and other factors such as socio-economic status [33,34,35,36], cultural factors [9] and other diseases [37]. Although GIS spatial analysis for epidemiological research provides a new tool, it cannot replace conventional monitoring methods. Future research techniques will combine comprehensive analysis of geographical and social factors for epidemic prevention.

In summary, hepatitis B infection and hepatoma remain public health problems in Shenzhen as in much of the developing world [25]. Based on the analyses in this study, we can determine the spatial distribution characteristics and use them to take measures toward disease prevention and control. Many different statistical analyses are also available to test for spatial disease clustering, with different powers for detecting different types of clustering [38]. GIS applications related to health have been introduced and used in environmental health [39,40,41,42], the quantification of environmental hazards [43,44,45,46], policy and planning [47,48,49,50,51]. Further investigation of these findings will hopefully help reduce hepatitis B infection and hepatoma incidence density and rates and can provide suggestions for public officials and policymakers for more effective allocation of sparse resources toward disease containment and prevention [38,52].
